# Barriers and corridors of gene flow in an urbanized tropical reef system

**DOI:** 10.1111/eva.13276

**Published:** 2021-07-27

**Authors:** Lutfi Afiq‐Rosli, Benjamin John Wainwright, Anya Roopa Gajanur, Ai Chin Lee, Seng Keat Ooi, Loke Ming Chou, Danwei Huang

**Affiliations:** ^1^ Department of Biological Sciences National University of Singapore Singapore Singapore; ^2^ Tropical Marine Science Institute National University of Singapore Singapore Singapore; ^3^ Yale‐NUS College National University of Singapore Singapore Singapore; ^4^ Centre for Nature‐based Climate Solutions National University of Singapore Singapore Singapore

**Keywords:** connectivity, coral reefs, genome‐wide, marine protected areas, population genomics, Scleractinia, SNPs

## Abstract

Information about the distribution of alleles among marine populations is critical for determining patterns of genetic connectivity that are essential in modern conservation planning. To estimate population connectivity in Singapore's urbanized equatorial reef system, we analysed single nucleotide polymorphisms (SNPs) from two species of reef‐building corals with distinct life histories. For *Porites* sp., a broadcast‐spawning coral, we found cryptic lineages that were differentially distributed at inshore and central‐offshore sites that could be attributed to contemporary surface current regimes. Near panmixia was observed for *Pocillopora acuta* with differentiation of colonies at the farthest site from mainland Singapore, a possible consequence of the brooding nature and relatively long pelagic larval duration of the species. Furthermore, analysis of recent gene flow showed that 60–80% of colonies in each population were nonmigrants, underscoring self‐recruitment as an important demographic process in this reef system. Apart from helping to enhance the management of Singapore's coral reef ecosystems, findings here pave the way for better understanding of the evolution of marine populations in South‐East Asia.

## 1 INTRODUCTION

Tropical coral reefs are in rapid decline due to climate change impacts and multiple local‐scale stressors (Cinner et al., 2018; Hoegh‐Guldberg et al., 2007; Hughes, Barnes, et al., 2017). The increasing magnitude and frequency of mass coral bleaching events have resulted in unprecedented mortality on many reefs (Heron et al., 2016; Hughes, Anderson, et al., 2018; Hughes, Barnes, et al., 2017) to the point that returning to their past configurations may no longer be possible (Hughes, Kerry, et al., 2017, 2018; Hughes et al., 2019). Although recovery of coral abundance, diversity and reef ecological functioning is conceivable in certain cases (Buglass et al., 2016; Cunning et al., 2016; Gilmour et al., 2013; Pisapia et al., 2016), successful recruitment of coral larvae from less impacted reef areas is key (Holbrook et al., 2018). However, larval dispersal capacities of corals at and surrounding affected sites are often unknown or not prioritized by resource managers during conservation planning (Balbar & Metaxas, 2019; McCook et al., 2009). In fact, a recent review found that only 11% of 739 marine protected areas (MPAs) have considered demographic or genetic connectivity as an ecological criterion, risking lower resilience and recovery chances for coral reefs (Balbar & Metaxas, 2019). This knowledge gap also hinders informed interventions such as temporary site closures to aid recovery, even in areas with strong legal protection.

Coral populations are genetically connected through the exchange of coral recruits over multiple generations, and the degree of connectivity can be inferred by comparing allele frequencies between populations (Selkoe et al., 2016). A recent analysis of the common coral *Acropora millepora* revealed very low levels of genetic divergence along the Great Barrier Reef (GBR), suggesting high gene flow through exchange of coral propagules (Matz et al., 2017). Conversely, populations with high disparity in allele frequencies are poorly connected and exchange few to no coral recruits over time, which can be attributed to ancient vicariance events or contemporary dispersal barriers (Lohman et al., 2011). For example, late‐Miocene coral reef populations in the Tropical Eastern Pacific (TEP) were well connected to the wider Caribbean reefs prior to the early‐Pliocene closure of the Isthmus of Panama, which has since become a dispersal barrier between these populations (Knowlton et al., 1993; Lessios, 2008; O'Dea et al., 2016). Relatedly, modern‐day TEP remains poorly connected to the Central Tropical Pacific (CTP) due to the Eastern Pacific Barrier that acts as a contemporary dispersal barrier (Baums et al., 2012; Romero‐Torres et al., 2018). Further, the broadcast‐spawning coral *Mussismilia hispida* can be divided into five genetically differentiated populations along the Southwestern Atlantic—consistent with present‐day oceanographic current patterns, zones of upwelling and historical sea‐level changes (Peluso et al., 2018). At much smaller spatial scales, physical characteristics such as the prevailing tidal magnitude can be important drivers of genetic differentiation. For example, reefs off the Kimberley coast of north‐western Australia that are experiencing higher tidal heights have greater connectivity with the metapopulation, and their population dynamics are generally influenced by the strong oceanographic currents in the region (Underwood et al., 2020). Estimates of genetic connectivity can aid in understanding larval dispersal patterns, which are otherwise impractical to track across a complex reef system (von der Heyden et al., 2014). This information can be incorporated into the design of MPAs and for defining management areas to help ensure continuous larval supply, enhancing the capacity of populations to recover from disturbances (Berumen et al., 2012; Hughes et al., 2010; Van Oppen & Gates, 2006) and improving the effectiveness of a broad range of conservation interventions (Christie et al., 2010; Magris et al., 2018). For instance, Bonin et al. (2016) highlighted the resilience of anemonefish populations at the Keppel Islands (GBR) that are supplied and maintained by recruits from distant source populations even if local breeders are lost. Additionally, Hock et al. (2017) showed that 112 reefs in the GBR have sufficient dispersal ability to facilitate recovery of disturbed areas, providing evidence for systemic resilience within the large reef system. In some cases, self‐recruitment within a population might be more prevalent on ecological timescales (over just a few generations) even though it may be well connected to other populations over evolutionary time (Christie et al., 2010). Indeed, because larvae of many coral species develop to allow high levels of self‐recruitment (Figueiredo et al., 2013), there can be two opposing influences on genetic diversity and resilience—adaptation when local environments differ, and reduction in genetic diversity in the absence of exogenous genetic exchange (Underwood et al., 2018). Given these effects, it is important to consider self‐recruitment patterns when assessing the genetic connectivity of reefs.

Advances in DNA sequencing technology and bioinformatics now permit the analysis of thousands of genetic loci, so previously unresolved patterns of genetic connectivity are now coming to light and can support conservation goals (Beltrán et al., 2017; Lopez et al., 2018). Earlier work on Singapore reefs using seven microsatellite loci found panmixia in a broadcast‐spawning coral *Platygyra sinensis* (Tay et al., 2015), a possible consequence of specific life history characteristics or limited resolution in the markers used (Durante & Baums, 2017). More resolved patterns of connectivity and gene flow can help improve current biodiversity management and conservation practice. For instance, if two populations are not well connected, it is preferable that they are treated as distinct management units (Eastwood et al., 2016). Among highly connected populations, source sites and sites with high levels of self‐recruitment should be prioritized for conservation (Jones et al., 2007; Krueck et al., 2017; Lequeux et al., 2018).

In this study, our aim is to assess fine‐scale (~180 km^2^) population genetic connectivity of two common reef‐building corals *Porites* sp. and *Pocillopora acuta* in Singapore using genome‐wide single nucleotide polymorphisms (SNPs). These two species have distinct reproductive modes (broadcast spawning and brooding, respectively; Kerr et al., 2011; Poquita‐Du et al., 2019). Variations in geographic connectivity patterns could thus be explained partly by their evolved life history strategies (Thomas et al., 2020; Underwood et al., 2020). Furthermore, these two species also have different dispersal capacities whereby *Poc*. *acuta* larvae can survive twice as long in its pelagic state compared with *Porites* sp. (Polato et al., 2010; Richmond, 1987). Integrating insights from multiple species—especially those with different life histories and dispersal capacities—will provide more robust inferences on population connectivity compared with the use of a single species (Magris et al., 2016). More broadly, our findings are expected to help enhance the management of Singapore's coral reefs and lay a foundation for clearer understanding of the evolution of coral populations in South‐East Asia.

## 2 MATERIALS AND METHODS

### 2.1 Study location and sampling

**FIGURE 1:**
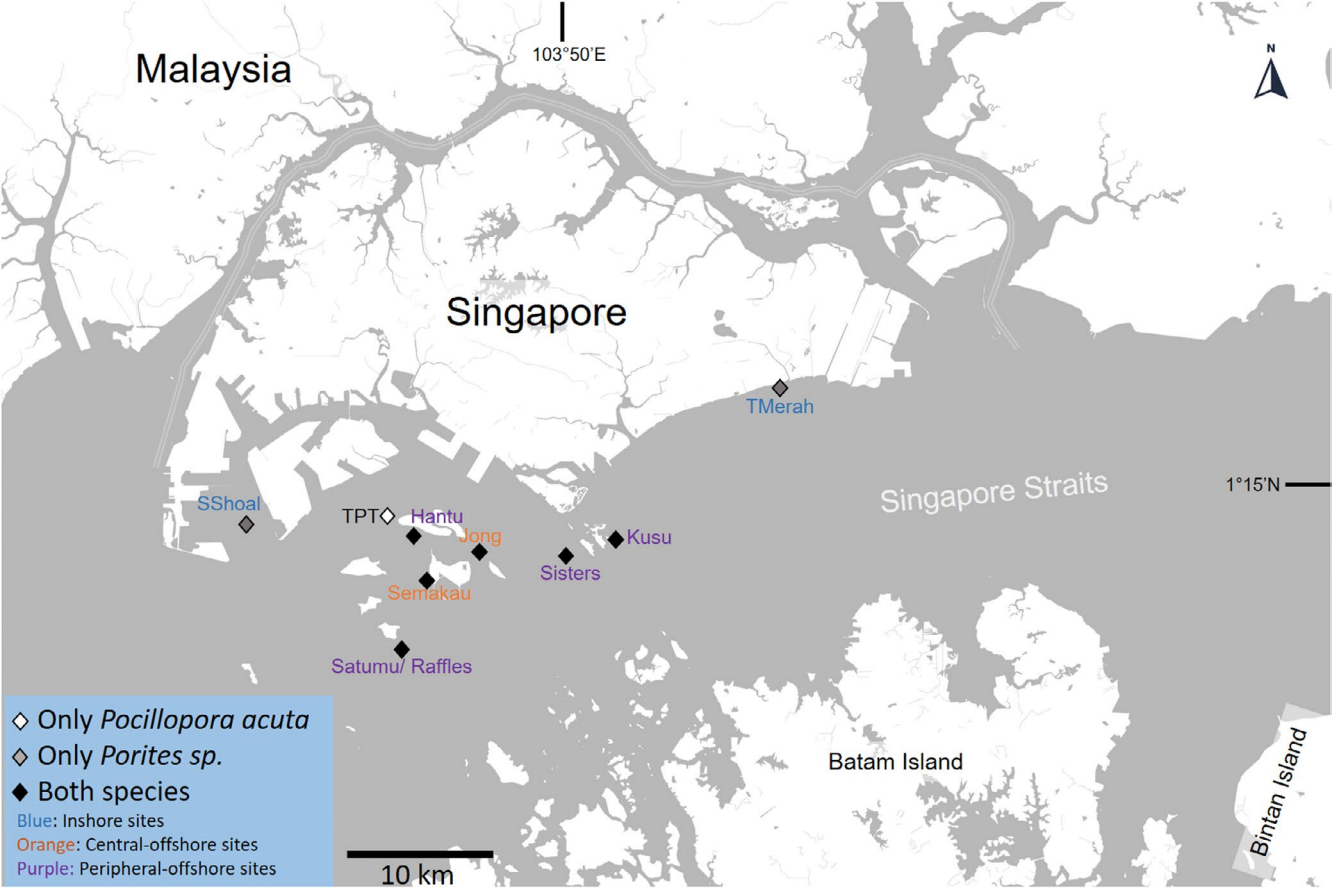
Sampling map for *Porites* sp. and *Pocillopora acuta* corals in Singapore's coral reef systems

We focused on the highly urbanized reef system in Singapore where we targeted seven to eight sampling sites depending on the abundance and availability of *Porites* sp. and *Pocillopora acuta* (Figure 1; see Table 1 for details on species sampling and identification and File S1 for morphometric analyses of *Porites* sp.). All samples were preserved in 100% molecular grade ethanol and stored at −80℃.

**TABLE 1:** Sampling sites and number of samples. Identification of *Pocillopora acuta* was performed *in situ* following Poquita‐Du et al. (2017); *Poc*. *acuta* was the only Pocilloporidae species remaining in Singapore (Poquita‐Du et al., 2019). Identification of *Porites sp*. required additional examination and verification (see File S1)

Site	*Porites sp*.	*Pocillopora acuta*
Hantu	20	4
Jong	20	12
Kusu	20	20
Satumu/ Raffles	20	20
Semakau	20	15
Sisters	20	20
Sultan Shoal	20	–
Tanah Merah	20	–
TPT	–	7
Total	160	98

### 2.2 DNA extraction, library preparation and sequencing

Genomic DNA was extracted on the abGenix™ automated nucleic acid extraction system (AITbiotech Pte Ltd) following the manufacturer's animal tissue genomic DNA extraction kit protocol. NextRAD genotyping‐by‐sequencing libraries (SNPsaurus, LLC) were prepared as in Russello et al. (2015). Briefly, DNA (~10 ng) was first fragmented and adapter‐ligated with the Nextera DNA Library Prep Kit (Illumina, Inc.). Fragmented DNA was then amplified for 26 cycles at 73℃, with one of the primers matching the adapter and extending nine nucleotides into the genomic DNA with the selective sequence 5′‐GTGTAGAGG‐3′. Thus, only fragments starting with a sequence that can be hybridized by the selective sequence of the primer would be efficiently amplified. The nextRAD libraries were sequenced on one HiSeq 4000 lane for 150‐bp single‐end reads (University of Oregon).

### 2.3 Genotyping and quality control

A sample of 1000 randomly selected reads from each individual sample was isolated and searched using BLASTn against the NCBI nucleotide database. Species information of the best BLAST hit was subsequently collected and used to check for contamination from bacteria, Symbiodiniaceae and other nontarget taxa (see File S1). The genotyping analysis used custom scripts (SNPsaurus, LLC) that trimmed the reads using bbduk (BBTools package; Brian Bushnell, Walnut Creek, CA, USA) (see File S1). Next, all remaining reads were mapped to the respective *Porites lutea* genome (from Liew et al., 2016: http://reefgenomics.org) and *Poc*. *acuta* de novo‐assembled reference (see File S1). Genotype calling was performed using Samtools and bcftools (Li et al., 2009) and compiled in Variant Call Format (VCF) files using custom parameters (see File S1). The VCF files were filtered to remove alleles with a population frequency of less than 3%. Loci that were heterozygous in all samples or had more than two alleles in a sample (suggesting collapsed paralogs) were also removed. PGDSpider (v2.1.1.5) (Lischer & Excoffier, 2012) was used to reformat the VCF files for downstream analyses. The remaining SNPs were evaluated for significant deviations from the Hardy–Weinberg equilibrium and linkage using arlecore (v3.5.2.2), with SNPs that deviated (excess or deficit) in more than five a priori populations removed as in Bongaerts et al. (2017). The *clonecorrect* function in R package *poppr* (v2.8.1) (Kamvar et al., 2014, 2015) was used to remove potential clones from the data set, with clonal groups reduced to a single representative per population. Finally, only SNPs with <1% missing data and samples with <15% missing data were retained to ensure high‐quality downstream analyses.

### 2.4 Data analyses

Three data sets with varying filtering parameters were assembled for analysis: overall data set (all loci), neutral data set (loci under selection removed) and outlier data set (only loci identified as under selection). BayeScan (v2.1) (Foll & Gaggiotti, 2008) using default parameters (see File S1) and Bayes factor cut‐off of 0.05 were used to identify loci under possible selection.

To assess genetic structure for each data set, Bayesian clustering analysis was performed in STRUCTURE v. 2.3.4 for up to eight possible genetic clusters (K) according to the total number of collection sites for each species. We considered correlated allele frequencies in the admixture model, using sampling locations as priors, and ran 10 iterations of 100,000 MCMC repetitions with 10,000 burn‐in period (Gilbert et al., 2012; Janes et al., 2017). MCMC convergence, where α values reached equilibrium, was examined using the *Data plot* option in STRUCTURE (Porras‐Hurtado et al., 2013). Variation of K values was then summarized and plotted in CLUMPAK (Kopelman et al., 2015). The optimal K was determined by examining the Ln Pr(X|K) and ΔK plots (Evanno et al., 2005; Janes et al., 2017; Pritchard & Wen, 2003). Principal component analysis (PCA) was performed in the R package *SNPRelate* v. 1.18 and *adegenet* v.2.1.3 (Jombart, 2008) to identify clusters without relying on population genetic models (Jombart et al., 2010).

We estimated contemporary gene flow in BayesAss v.3.0.4 (Wilson & Rannala, 2003) using 10,000,000 MCMC repetitions, a burn‐in of 1,000,000 and a sampling interval of 1,000 iterations. Parameters for allele frequencies (ΔA) and inbreeding coefficients (ΔF) were adapted to 0.30 to improve mixing of the chains (Winter et al., 2018). Convergence was checked in Tracer v.1.7.0 (Rambaut et al., 2018) and by result consistency over 10 runs with random initial seeds. As estimates of gene flow may be biased when analysing individuals with different ancestries in the same data set (Pante et al., 2015), we ran a separate BayesAss analysis on each genetic lineage identified by both STRUCTURE analysis and PCA. Results were visualized for each lineage using Circos plots using the package *circlize* v. 0.4.12 and *tidyverse* v.1.3.0 in R (Gu et al., 2014; Holland et al., 2017).

An individual‐based analysis that relies on detecting deviations from the isolation‐by‐distance (IBD) models (Keis et al., 2013; Tang et al., 2018) was used to characterize barriers of and corridors for dispersal of each species using R package ResDisMapper (Tang et al., 2019). First, distributions of genetic distance (Nei's standard genetic distance) and geographic distance (both measured in GenAlEx v 6.5) were checked using two modelling methods—linear and nonlinear—before a best‐fit method based on *R* ^2^ value was chosen for IBD residual calculation for each pair of individuals. Resistance values, together with their corresponding statistical significance over the landscape, were then calculated using default settings.

## 3 RESULTS

**FIGURE 2:**
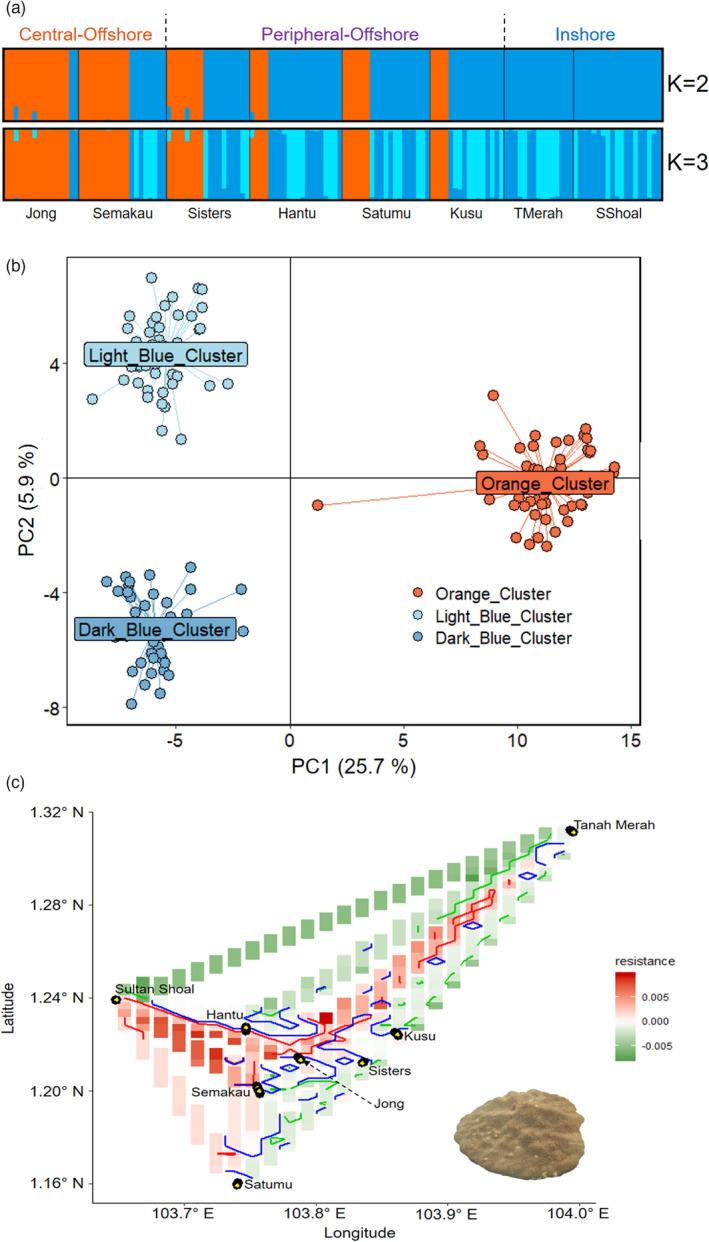
Genetic structure of *Porites* sp. based on (a) STRUCTURE and (b) principal component analysis. (c) Resistance map produced by ResDisMapper. Areas with resistance values that are higher/lower than those from a null distribution with high probability, and lie within the red/green contours represent a significant barrier/corridor. Areas within the blue contours have resistance values with high probability of being positive or negative (high ‘certainty’). Yellow circles indicate sampling points

**FIGURE 3:**
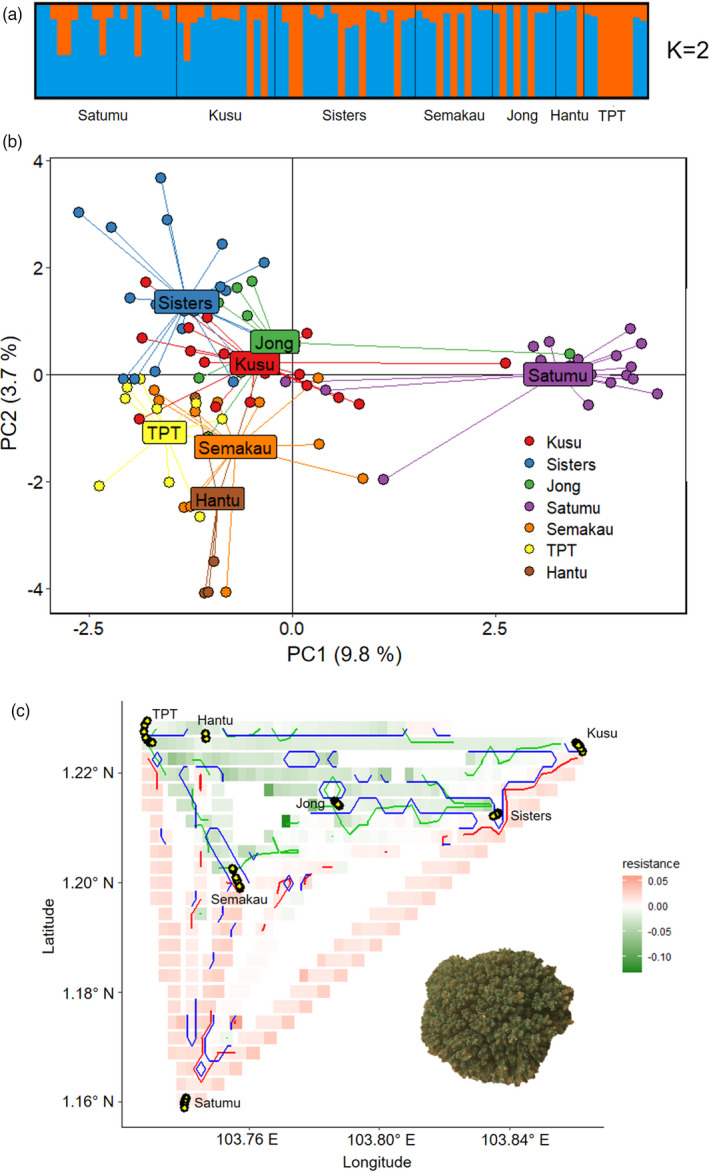
Genetic structure of *Pocillopora acuta* based on (a) STRUCTURE and (b) principal component analysis. (c) Resistance map produced by ResDisMapper. Areas with resistance values that are higher/lower than those from a null distribution with high probability, and lie within the red/green contours represent a significant barrier/corridor. Areas within the blue contours have resistance values with high probability of being positive or negative (high ‘certainty’). Yellow circles indicate sampling points

Sequencing of the nextRAD libraries resulted in an average of ~1.7 million reads per sample that mapped to the references (*n* = 258). Genotype calling initially yielded 36,836 biallelic single nucleotide polymorphisms (SNPs) for *Porites* sp. (*n* = 160) and 28,188 biallelic SNPs for *Pocillopora acuta* (*n* = 98). After quality control to remove loci that were linked, deviate from the Hardy–Weinberg equilibrium or had more than 1% missing data per loci, removing colonies with more than 15% missing data per sample, and following minimal representation filtering, we retained 3649 biallelic SNPs for *Porites* sp. (*n* = 149) and 5846 biallelic SNPs for *Poc*. *acuta* (*n* = 88). *clonecorrect* (Kamvar et al., 2014) under default parameters did not identify any clones in either species. BayeScan identified 33 and 26 outlier SNPs for *Porites* sp. and *Poc*. *Acuta*, respectively. STRUCTURE analysis with or without SNPs identified as putatively under selection did not alter assignments of individuals to clusters (Figures 2 and 3), and because of this, we opted to use the larger dataset for subsequent analyses.

Evanno's method showed that the most likely ‘K’ is two for *Porites* sp. (Figure S8 in File S1). However, K = 3 is also plausible given the ancestry assignments in the STRUCTURE plots (Figure 2A; Figure S6 in File S1). At K = 2, STRUCTURE plots exhibited population structuring between the Blue (Dark Blue and Light Blue) and Orange clusters (Figure 2A). These cluster assignments can be attributed to the inshore sites with colonies primarily from the Blue cluster (Tanah Merah and Sultan Shoal), and the central‐offshore sites with colonies primarily from the Orange cluster (Semakau and Jong). The peripheral‐offshore sites (Kusu, Satumu, Sisters and Hantu) is mainly constituted of genetic assignments from both Orange and Blue clusters (Figure 2A). The same pattern can be seen in our PCA plot (Figure 2B). At K = 3, the Blue cluster further separated into two subclusters (Figure 2A)—Dark Blue and Light Blue—albeit with lower distinctiveness (5.9% on PC2; Figure 2B) compared with the separation between the Orange and Blue clusters (25.7% on PC1; Figure 2B). Independent analysis of each cluster suggested panmixia within clusters (Figure S10 in File S1).

**FIGURE 4:**
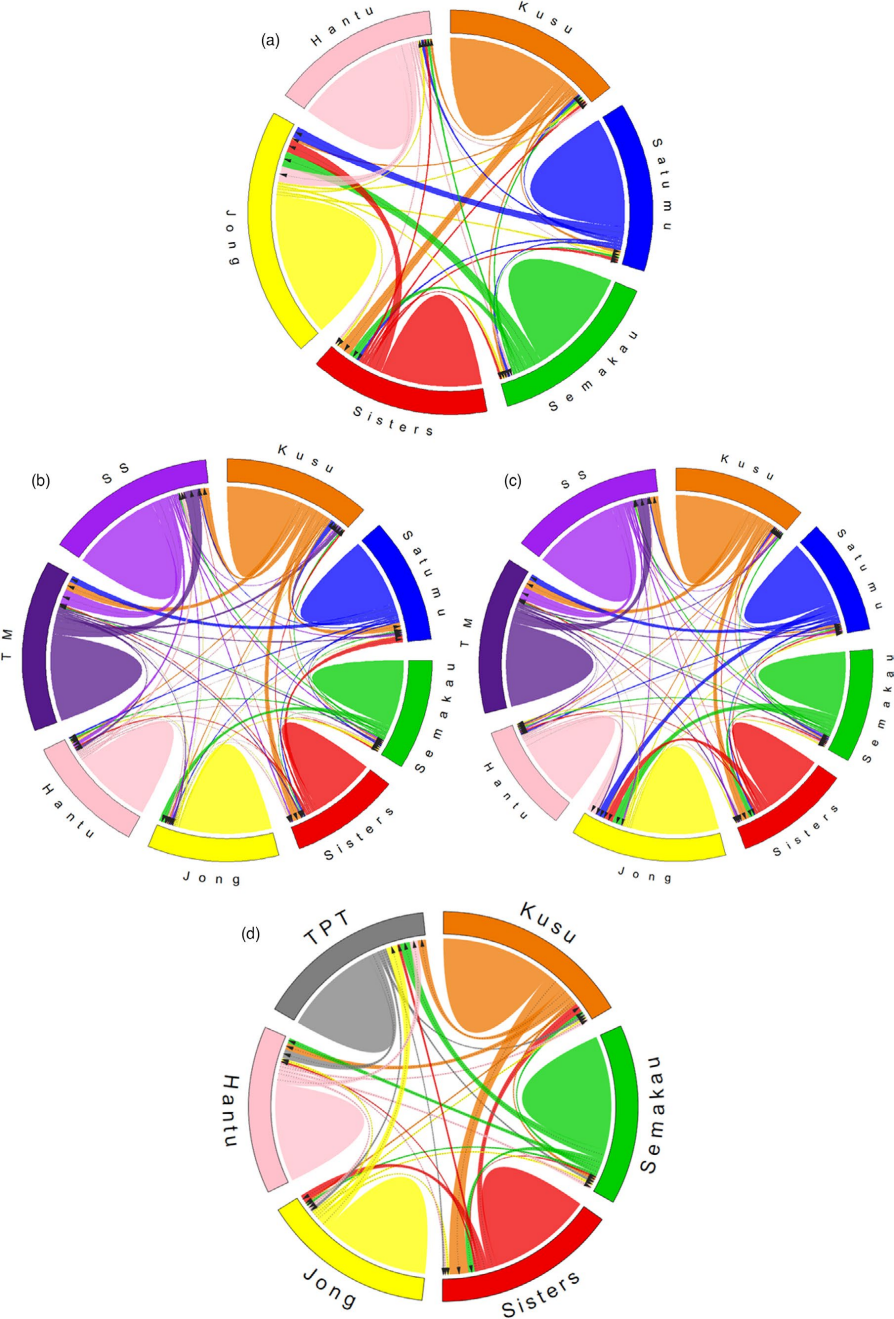
Circos plots of contemporary gene flow estimation with BayesAss v.3.0.4 for (a) Orange cluster of *Porites* sp., (b) Light Blue cluster of *Porites* sp., (c) Dark Blue cluster of *Porites* sp. and (d) *Pocillopora acuta*. Arrows represent directionality of migrant movement (refer to Tables S6–S7 in File S1 for actual values)

BayesAss analyses showed that self‐recruitment was high at all sites for all three clusters (~60%–80% of nonmigrants) (Figure 4; Table S6A–C in File S1). Strong evidence of migrant exchange (10%–12% migrant movement) was detected from Kusu to Sisters and Semakau to Jong for the Orange cluster (Figure 4A,B;Table S6A in File S1). For both Dark Blue and Light Blue clusters, strong evidence of migrant exchange (10%–12% migrant movement) was also recorded among the inshore sites (Tanah Merah and Sultan Shoal), while moderate migrant movement (5%–9% of migrants movement) was detected from Kusu to at least three other sites (Figure 4B; Table S6B,C in File S1). The relatively high level of inshore migrant exchange was supported by results from ResDisMapper, where a dispersal corridor was identified along the southern coast of mainland Singapore (Figure 2C).

Evanno's method and STRUCTURE showed no separation into distinct clusters for *Poc*. *acuta* (Figure 3A), while PCA supported differentiation between Satumu and the other islands (Figure 3B). BayesAss analysis performed on all sites except Satumu showed a high prevalence of self‐recruitment at all sites (60%–80%) and yielded strong evidence of migrant exchange (10%–12% migrant movement) from Kusu to Sisters (Figure 4D; Table S7 in File S1). Similarly, ResDisMapper indicated corridors to dispersal among all nearshore sites, that is all sites apart from Satumu, which faced barriers to dispersal to and from all other sites (Figure 3C).

## 4 DISCUSSION

The ability of a reef to recover following disturbance relies upon supply of migrants from surrounding areas (Berumen et al., 2012). However, the extent of reef connectivity particularly in a highly disturbed reef system has not previously been examined using genome‐wide markers. Here, we highlight the presence of cryptic lineages, subtle fine‐scale population differentiation and predominance of self‐recruitment on the highly sedimented equatorial reef system of Singapore. In particular, we found that *Porites* sp. corals at inshore sites (Tanah Merah and Sultan Shoal) were distinct from central‐offshore sites (Semakau and Jong). This pattern was supported by relatively strong migrant exchange and dispersal corridors among inshore sites and separately among central‐offshore sites. *Pocillopora acuta* maintained a panmictic population across all sites except for Satumu, the farthest reef from mainland Singapore.

Cryptic diversity is prevalent in the marine environment (Appeltans et al., 2012), especially in locations with novel substrates, which may reduce gene flow or influence recruitment of different genotypes (Chang et al., 2018; Simon et al., 2020). This issue is important to address as failure to recognize boundaries of evolutionarily relevant units may lead to biased estimates of connectivity (Pante et al., 2015). For corals, extensive taxonomic and phylogenetic studies are ongoing (Kitahara et al., 2016). There is evidence suggesting that cryptic species occur in particular coral taxa (Schmidt‐Roach et al., 2014; Torda et al., 2013), including the genus *Porites* (Forsman et al., 2015). Yet, there are also studies based on integrative morpho‐molecular analyses showing that some seemingly distinct morphospecies actually belong to a single species (Pinzón et al., 2013; Stefani et al., 2011; Terraneo et al., 2019). While *Poc*. *acuta* in Singapore has been investigated thoroughly and deemed to be a single species (2017, 2019), there have been at least two co‐occurring massive *Porites* morphotypes recorded here (Chow et al., 2019; Huang et al., 2009; Wong et al., 2018).

Our morphometric analyses showed while both types of ventral triplet formation—fused and free for *Por*. *lutea* and the closely related *Por*. *lobata*, respectively—were found in our samples, they did not form distinct clusters (Figures S3–S5 in File S1). To investigate this further, we performed separate STRUCTURE analyses for colonies with fused and free triplet formations using parameters outlined above. These additional results showed that (1) genetic differentiation among samples was not caused by possible species separation associated with the differing triplet forms (Figure S9A in File S1), and (2) the underlying population dynamics observed for *Porites* sp. were consistent with separate analyses for fused and free morphotypes (Figures S9B,C in File S1). STRUCTURE analysis for all *Porites* sp. colonies also revealed the presence of cryptic genetic lineages (Figure 2A), which were not discernible by morphology. Therefore, we further analysed the three putative lineages separately to estimate contemporary migrant movement using BayesAss (Figure 4), yielding results that were consistent with the combined analysis and with estimates for the fused and free morphotypes.

The differentiation pattern of *Porites* sp. observed here differs from previous results obtained using microsatellite loci for another broadcast‐spawning coral, *Platygyra sinensis*, within Singapore waters (Tay et al., 2015). The latter was shown to be a highly connected, panmictic population. This variation could be due to either the limited resolution of the microsatellite markers used for *Pla*. *sinensis* or the fact that *Porites sp*. is a gonochore while *Pla*. *sinensis* is hermaphroditic (Durante & Baums, 2017). A SNP‐based study of broadcast‐spawning hermaphrodites would clarify this pattern, but it is already clear that small differences in reproductive traits can have dramatic impacts on population connectivity (Holland et al., 2017), whereby broadcasting gonochores tend to have limited connectivity compared with the hermaphrodites (Eckert et al., 2019; Rippe et al., 2017; Shinzato et al., 2015; Thomas et al., 2020).

The distribution of cryptic lineages between the inshore—predominantly the Blue cluster—and central‐offshore sites—predominantly the Orange cluster—in *Porites* sp. can be explained by contemporary surface current regimes. During the spawning and postspawning months of April to June, residual surface currents along the Singapore Strait oscillate between west to east and east to west (Sin et al., 2016; Video S2). This pattern persists throughout the South‐west Monsoon (June to September), resulting in relatively lower current velocities around the Southern Islands complex where the reefs are situated (Figure 1), with the main flow occurring along the deeper waters of the Singapore Strait (Video S3). Lower current velocities close to the centre of the reef complex may generate a weak dispersal barrier and promote the retention of propagules within the central‐offshore sites.

Inshore–offshore differentiation over short distances has also been shown in other reef systems. For example, Tisthammer et al. (2019) highlighted genetic differentiation in *Porites lobata* between nearshore and offshore sites that were only less than 2 km apart due to distinct water quality and sedimentation load. Such environmental divergence was not as apparent in the present study since the waters along the southern coast of Singapore are homogeneous and well mixed (Sin et al., 2016; Wainwright et al., 2019), underscoring the importance of understanding surface current regimes and the complexity in interpreting connectivity patterns in general.

For *Poc*. *acuta*, the PCA plot and characterization of barriers and corridors to dispersal showed differentiation between Satumu and the remaining sites despite STRUCTURE showing no distinct clusters. These results suggest panmixia among sites other than Satumu, the farthest site from mainland Singapore. *Pocillopora acuta* releases planula larvae every month (Poquita‐Du et al., 2017) and has a relatively long pelagic larval duration (twice as long when compared to *Porites* sp.) (Polato et al., 2010; Richmond, 1987). Consequently, the close proximity of reefs to one another and net east‐to‐west water movement (Sin et al., 2016; Tay et al., 2012; 2015) can disperse the monthly supply of planulae throughout the reef system and drive the high genetic connectivity of *Poc*. *acuta* in Singapore waters. This result is unexpected given that brooders are usually considered to have limited dispersal range (Serrano et al., 2016). However, brooding species have shown variable patterns of genetic differentiation in other areas. For instance, work on *Poc*. *verrucosa* highlighted panmixia across different environmental conditions in the Red Sea based on microsatellite markers (Robitzch et al., 2015), while similar analyses on *Poc*. *damicornis* revealed strong genetic differentiation among sites in Madagascar (Gélin et al., 2018). A recent genomic analysis on the brooding coral *Isopora brueggemanni* in Western Australia also found strong genomic differentiation (Thomas et al., 2020). Aside from geographic patterns, genomic differentiation was shown to occur across a depth gradient for *Agaricia fragilis* in Bermuda (Bongaerts et al., 2017). These variable patterns emphasize the complexity associated with inferring population structure and demonstrate the need for studying multiple species to fully understand genetic connectivity in the marine environment (Palumbi, 1994).

Our analyses suggest that self‐recruitment is a key demographic process in Singapore's coral reef system. This finding is unsurprising despite contrary early ideas (Lequeux et al., 2018; Wood et al., 2014) given that population replenishment of some highly dispersive taxa have been shown to be dominated by self‐recruitment (Berumen et al., 2012; Wainwright, Afiq‐Rosli, et al., 2019; Wainwright et al., 2018, 2019; Wainwright, Zahn, et al., 2019). For many coral species, routine dispersal is estimated to be around 20–30 km, but it can be as limited as a few hundred metres (see Underwood et al., 2018). Constrained dispersal potentially leads to either local adaptation when reef environments differ, or reduction in genetic diversity when gene flow is curtailed (Underwood et al., 2018). As we found no difference in genetic structure when putatively adaptive loci were used (Figure S5 in File S1), and because the marine environment of Singapore's Southern Islands is homogeneous (Sin et al., 2016; Wainwright, Afiq‐Rosli, et al., 2019), the latter case appears more likely. However, we suggest that there is sufficient contemporary gene flow across sites to prevent the reduction in genetic diversity. This inference would be in concordance with the one migrant per generation (OMPG) rule, which states that just one migrant per generation is adequate to prevent loss of genetic diversity (Greenbaum et al., 2014; Wang, 2004). Further, despite the predominance of self‐recruitment, panmictic populations are still possible over multiple generations if there is occasional long‐distance dispersal. Indeed here, we show that at least 40% of individuals at most sites were composed of migrants, contributing to the underlying genetic structuring of both *Porites* sp. and *Poc*. *acuta*.

The ability to recover after a disturbance depends on the availability of larvae and successful recruitment. Unfortunately, coral spat settlement in Singapore is depressed (Bauman et al., 2015). Thus, it is essential to identify source populations within Singapore's reef and protect them accordingly to maximize larval supply. Our BayesAss analyses suggest that Kusu is a source site for the peripheral‐offshore and all the nearshore sites for *Porites* sp. and *Poc*. *acuta*, respectively. Kusu's eastward location also increases its chances of receiving larval flow from healthy reefs outside Singapore waters. Therefore, Kusu is the best candidate among all study sites for a marine protected area and will complement the established Sisters’ Islands Marine Park as a major source of biodiversity for Singapore's reef system (Jaafar et al., 2018). Indeed, a previous study on *Platygyra sinensis* based on microsatellites also suggested Kusu as a potential target for conservation due to its high genetic diversity of corals (Tay et al., 2015). In general, BayesAss results should be interpreted with caution due to issues such as nonconvergence (Meirmans, 2014) and loss of statistical power when certain assumptions such as linkage equilibrium are violated (Faubet & Gaggiotti, 2008). However, since we used unlinked loci and have ensured convergence in our BayesAss runs, these factors are unlikely to affect our general conclusions.

Given the highly degraded marine environment of Singapore and ongoing coastal development (Chou, 2006; Chou et al., 2019), genetic connectivity and diversity throughout its coral reefs are critical measures to consider during interventions. Reef restoration via coral gardening is used in Singapore to restore degraded and damaged reefs (Afiq‐Rosli et al., 2017), and it has been shown that genetic diversity of these coral transplants is an important determinant of success (Afiq‐Rosli et al., 2019; see also Shearer et al., 2009). Based on our results and the suggestion that it is important to transplant genotypes to their optimal environments (Drury et al., 2017), we suggest that transplantation of *Porites* sp. from the inshore sites to the central‐offshore sites and vice versa should not be done until genotypic and phenotypic (e.g. growth and survival) comparisons are performed. *Porites* sp. from both the inshore sites and central‐offshore sites can, however, be reared or transplanted in any of the peripheral‐offshore sites as both cryptic lineages are present there. For *Poc*. *acuta*, such restrictions might not be necessary since our results suggested panmixia with subtle genomic differentiation between Satumu and all other sites. Nonetheless, caution should be taken if *Poc*. *acuta* were to be reared or transplanted from or to Satumu to avoid maladaptation (Wainwright, Afiq‐Rosli, et al., 2019).

In the event of high coral mortality at neighbouring reefs (e.g. due to thermal stress and associated bleaching), high migrant movement from Singapore to these reefs would enhance their natural recovery potential. Likewise, high migrant movement from other reefs to Singapore would greatly enhance the latter's resilience against global and local disturbances, especially those related to coastal development. In any case, identifying reefs with high connectivity to Singapore's reef system is essential for its long‐term resilience. Tay et al. (2012) had suggested that reefs at the northern coast of Bintan Island, Indonesia, may be a potential larval source. However, it was later discovered that there are subtle genetic differentiation between the reefs of Bintan and Singapore, with the Singapore Strait acting as a mild gene flow barrier (Tay et al., 2015). The present study corroborates this inference as the Singapore Strait is subjected to strong current flow away from the Southern Islands complex (Videos S2 and S3). Further investigation is warranted to identify other potential larval sources for Singapore's reef system. A likely candidate is the group of islands off the east coast of Malay Peninsula as these reefs appear connected by currents that abut Singapore's southern coasts, favouring larval transport from these islands without having to cross the Singapore Strait.

Overall, this study has highlighted the presence of subtle fine‐scale genetic structure in the highly sedimented equatorial reefs of Singapore. In particular, inshore and central‐offshore populations of *Porites* sp. have low levels of connectivity between them and comprise distinct lineages, while *Pocillopora acuta* populations are highly connected with differentiation of the southernmost population. These patterns are a likely consequence of contemporary surface current regimes, with a general east‐to‐west flow, which disperses monthly *Poc*. *acuta* planulae throughout the reef system and lower current velocities close to the central‐offshore sites during spawning period that help retain *Porites* sp. propagules. Most critically, we show that self‐recruitment is an important demographic process for corals here and identify areas that may be larval sources and sites with high levels of self‐recruitment for enhanced management to protect the resilience of Singapore's reef system.

## CONFLICT OF INTEREST

None declared.

## Supporting information

File S1

Video S1

Video S2

## Data Availability

The data for this study are available at the Dryad Digital Repository: https://doi.org/10.5061/dryad.7sqv9s4sd
