# Supplementary material for: Barriers and corridors of gene flow in an urbanized tropical reef system
Source: Evol Appl. 2021 Jul 27;14(10):2502–15. doi: 10.1111/eva.13276 (PMC8549622; doi:10.1111/eva.13276)
Supplement: Supplementary file 1 — File S1 [file EVA-14-2502-s001.docx]

**Barriers and corridors of gene flow in an urbanised tropical reef system**

Lutfi Afiq-Rosli, Benjamin John Wainwright, Anya, Ai Chin Lee, Seng Keat Ooi, Loke Ming Chou, Danwei Huang

**Supplementary File 1**

**Specimen collection and imaging**

*Porites lutea* and *Por. lobata* are amongst the most common massive *Porites* species occurring in Singapore (Huang et al. 2009; Wong et al. 2018). Given that these two species and other massive species could be easily confused owing to their morphological similarities (Veron 2000), we performed a morphometric analysis to verify that all colonies analysed for population genomics here were of a single species.

We included in the analysis massive *Porites* colonies from seven sites in Singapore that most closely matched the description and images in Veron and Pichon (1982) and Veron (2000) (Table S1). A skeletal fragment ranging from 2.5×2.5 to 7×7 cm^2^ was chiseled off from the edge of each colony. For each site, 20 different colonies were sampled with a distance of at least 10 m between colonies to minimise the chance of collecting clone mates. Following subsampling for DNA, specimens were washed to remove the tissue and placed in a solution of equal parts water and household bleach (sodium hypochlorite, Clorox) for a week. The bleach was then washed off, and the samples were dried in the laboratory oven at 55°C for ~40 to 50 hours.

For each voucher specimen, four images were taken at different magnifications, at 0.78x, 1.0x, 1.25x and 4x, respectively, using a dissecting microscope. A monofilament line of known thickness (0.5 mm) was used to scale each image.

**Table S1.** Sampling sites and number of samples. Identification of *Pocillopora acuta* was performed *in situ* following Poquita-Du et al. (2017); *Poc. acuta* was the only Pocilloporidae species remaining in Singapore (Poquita et al. 2019). Identification of massive *Porites* sp. required additional examination and verification (see below); samples from Tanah Merah were not included in this analysis.

| **Site** | ***Porites* sp.** | ***Pocillopora acuta*** |
| --- | --- | --- |
| Hantu | 20 | 4 |
| Jong | 20 | 12 |
| Kusu | 20 | 20 |
| Satumu | 20 | 20 |
| Semakau | 20 | 15 |
| Sisters | 20 | 20 |
| Sultan Shoal | 20 | – |
| Tanah Merah | 20 | – |
| TPT | – | 7 |
| Total | 160 | 98 |

**Morphometric measurements and analysis**

Morphometric measurements followed a literature review of past taxonomic and morphometric studies performed on scleractinian corals, focusing on *Porites* (Forsman et al., 2015; Tisthammer & Richmond, 2018; Stefani et al., 2008; Knowlton et al., 1997; Brakel, 1977; Todd et al., 2004; Weil, 1992; López-Pérez, 2013; Jameson & Cairns, 2012). We chose the most commonly measured trait characteristics which were also taxonomically important and could be measured with high precision.

After the images were scaled, ImageJ version 1.51v (Schneider et al., 2012) was used to take the measurements shown in Figures S1, S2 and Table S2. For each colony, 5–10 corallites were measured depending on the number of observable corallites that were mature and fully formed. This sample size was deemed adequate to capture the within-sample variation (Tisthammer & Richmond, 2018). Measurements among corallites in each colony were averaged. Principal component analysis (PCA) on log-transformed data was conducted on 11 of the 18 quantitative characters after omitting highly-correlated traits, such as LL1 and LL2 as well as VL1, VL2 and VL3 (Table S3).


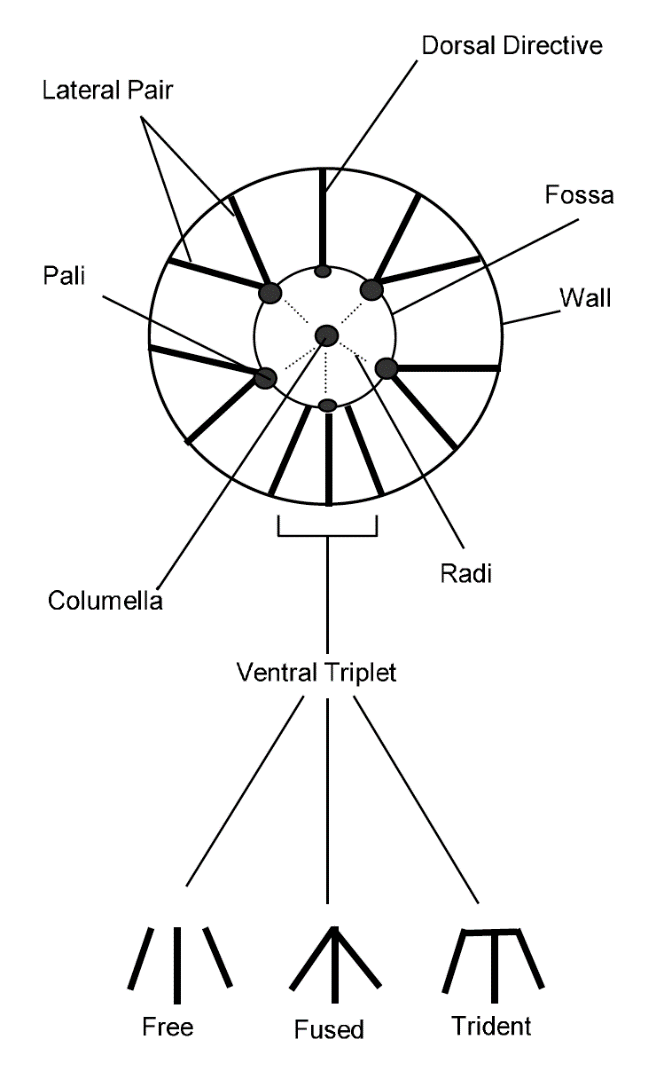


**Figure S1.** Illustration of the corallite morphometric characters used in this study, from Forsman et al. (2015).

**Table S2.** Skeletal characteristics used for morphometric analysis. Characters 1 to 16 are distance measurements in mm, characters 17 and 18 are area measurements in mm^2^.

| **No.** | **Character** | **Abbreviation** |
| --- | --- | --- |
| 1 | Dorsal length | DL |
| 2 | Dorsal width | DW |
| 3 | Lateral length 1 | LL1 |
| 4 | Lateral width 1 | LW1 |
| 5 | Lateral length 2 | LL2 |
| 6 | Lateral width 2 | LW2 |
| 7 | Ventral length 1 | VL1 |
| 8 | Ventral width 1 | VW1 |
| 9 | Ventral length 2 | VL2 |
| 10 | Ventral width 2 | VW2 |
| 11 | Ventral length 3 | VL3 |
| 12 | Ventral width 3 | VW3 |
| 13 | Corallite diameter 1 (length) | CD1 |
| 14 | Corallite diameter 2 (width) | CD2 |
| 15 | Fossa diameter 1 (length) | FD1 |
| 16 | Fossa diameter 2 (width) | FD2 |
| 17 | Corallite area | CA |
| 18 | Fossa area | FA |
| 19 | Triplet structure | – |


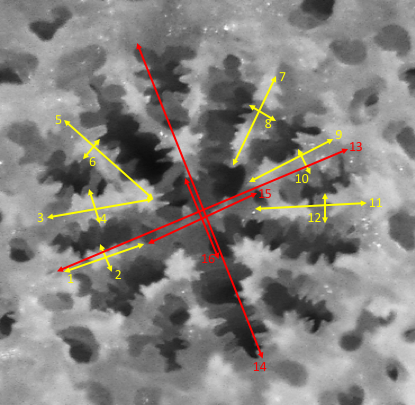


**Figure S2.** Skeletal characteristics used for morphometric analysis, according to Table S2.

**Table S3.** Minimum and maximum values of each of the morphometric characters used in the principal component analysis.

| **Character** | **Minimum value** | **Maximum value** |
| --- | --- | --- |
| Dorsal length (mm) | 0.1680 | 1.1350 |
| Dorsal width (mm) | 0.0466 | 0.3317 |
| Lateral length (mm) | 0.2352 | 1.3114 |
| Lateral width (mm) | 0.0451 | 0.3220 |
| Ventral length (mm) | 0.1577 | 1.1232 |
| Ventral width (mm) | 0.0485 | 0.3510 |
| Corallite diameter (mm) | 0.1094 | 0.7371 |
| Fossa diameter (mm) | 0.2438 | 1.1234 |
| Corallite area (mm^2^) | 0.3622 | 7.8609 |
| Fossa area (mm^2^) | 0.0510 | 1.2500 |
| Ratio of FA:CA | 0.0708 | 0.2188 |

Results of the PCA showed that the first principal component axis (PC1) explained 84.69% of the total variation while the second axis (PC2) explained 10.82% (Figure S3). Dorsal, lateral and ventral length and width, as well as corallite and fossa diameter and area were strongly covarying with PC1. PC2 was explained mainly by ratio of fossa to corallite area. Colonies from all sites overlap considerably with one another, and no single site contained colonies distinct from others (Figure S3).

Groups of colonies based on the type of ventral triplet formation (free, fused or trident) also largely overlapped in the morphospace. This is important as the main diagnostic feature for distinguishing *P. lutea* and *P. lobata* from each other is the triplet formation—the former having fused triplet margins and the latter possessing free margins (Veron 2000). Recent studies have supplemented critical observations (Forsman et al., 2015; Tisthammer & Richmond, 2018), especially with Forsman et al. (2015) characterising the fused margins in *P. lobata* to be closer to a trident formation. Our analysis found no distinct clusters comprising colonies with free or trident formation, highlighting that regardless of the diagnostic feature used to distinguish *P. lutea* from *P. lobata*, the colonies used here for population genomic analysis should be considered a single species.


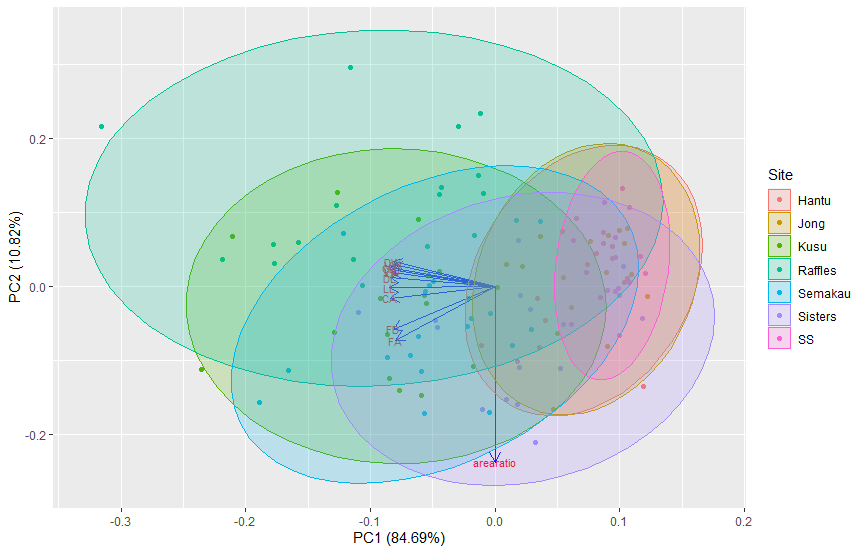


**Figure S3.** Principal component analysis based on 11 morphological characters of 119 *Porites* colonies grouped according to sampling sites, with corresponding 95% confidence ellipses. Samples from Tanah Merah were not included in this analysis.


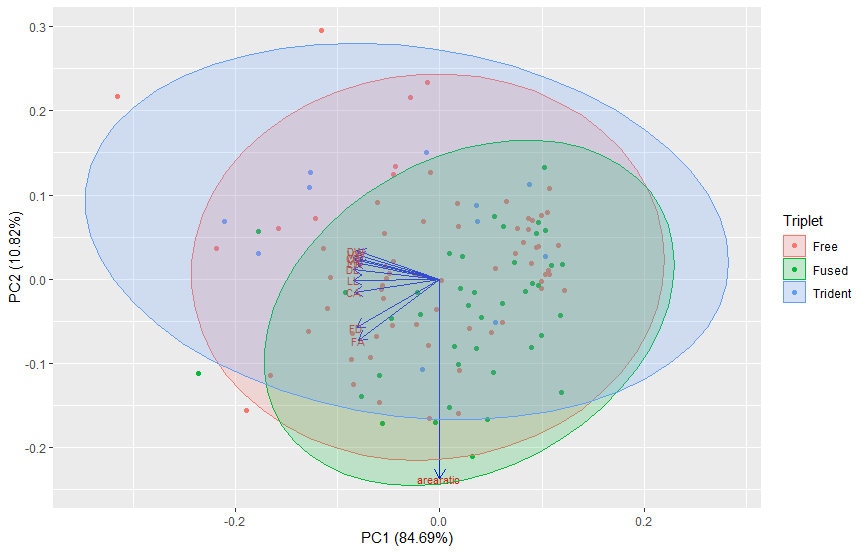


**Figure S4.** Results of principal component analysis based on 11 morphological characters of 119 *Porites* colonies grouped according to triplet formation observed for each colony (critical for species identification), with corresponding 95% confidence ellipses.


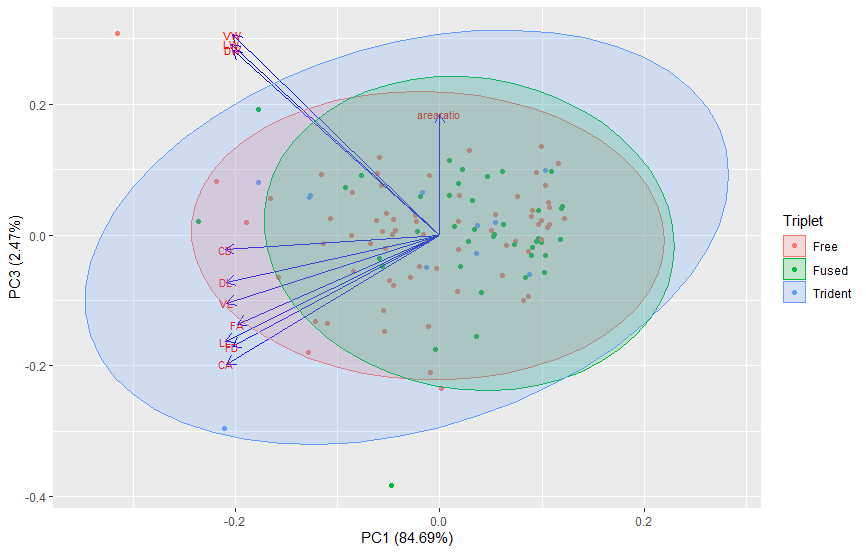


**Figure S5.** Results of principal component analysis; showing PC1 vs PC3; based on 11 morphological characters of 119 *Porites* colonies grouped according to triplet formation observed for each colony (critical for species identification), with corresponding 95% confidence ellipses.

**Genotyping of *Porites* sp.**

We used custom scripts (SNPsaurus, LLC) to trim the reads using bbduk (BBMap tools, <http://sourceforge.net/projects/bbmap/>):

bbmap/bbduk.sh in=reads/run_2135/2135_ATGCCGCT-CTAAGCCT_S161_L001_R1_001.fastq.gz out=reads/run_2135/2135_ATGCCGCT-CTAAGCCT_S161_L001_R1_001_t.fastq.gz ktrim=r k=17 hdist=1 mink=8 ref=bbmap/resources/nextera.fa.gz minlen=100 ow=t qtrim=r trimq=10

Next, all reads were mapped to the *Por. lutea* reference with an alignment identity threshold of 0.9 using bbmap (BBMap tools). Genotype calling was done using Samtools and bcftools (samtools mpileup -gu -Q 15 -t DP,DPR -f ref.fasta -b samples.txt | bcftools call -cv - > genotypes.vcf). The vcf was filtered to remove alleles with a population frequency of less than 3%. Loci were removed that were heterozygous in all samples or had more than 2 alleles in a sample (suggesting collapsed paralogs).

**Genotyping of *Pocillopora acuta***

We used custom scripts (SNPsaurus, LLC) to trim the reads using bbduk (BBMap tools, <http://sourceforge.net/projects/bbmap/>):
bbmap/bbduk.sh in=reads/run_2132/2132_CAAGTGTC-CTCTCTAT_S259_L002_R1_001.fastq.gz out=reads/run_2132/2132_CAAGTGTC-CTCTCTAT_S259_L002_R1_001_t.fastq.gz ktrim=r k=17 hdist=1 mink=8 ref=bbmap/resources/nextera.fa.gz minlen=100 ow=t qtrim=r trimq=10

Next, all reads were mapped to the P_acuta_refv1_1.fa reference with an alignment identity threshold of 0.9 using bbmap (BBMap tools). Genotype calling was done using Samtools and bcftools (samtools mpileup -gu -Q 15 -t DP,DPR -f ref.fasta -b samples.txt | bcftools call -cv - > genotypes.vcf). The vcf was filtered to remove alleles with a population frequency of less than 3%. Loci were removed that were heterozygous in all samples or had more than 2 alleles in a sample (suggesting collapsed paralogs).

The P_acuta_refv1_1.fa reference was created by sequencing sample Pa_SS_01 with WGS paired-end 150 bp reads, then assembling with abyss-pe with default parameters. The contigs were filtered to keep those of length 300 bp or greater, and then checked for contamination with blastn, removing bacteria matches.

**BayeScan analysis parameters**

BayeScan (v2.1) (Foll and Gaggiotti, 2008) using default and Bayes factor cut-off of 0.05 were used to identify loci under possible selection.

1. Parameters of chain: (a) sample size: 5,000; (b) thinning interval: 10; (c) pilot run: 20; (d) pilot run length: 10,000; (e) additional burn-In: 100,000
2. Parameter of the model: prior odds for neutral model: 1

**STRUCTURE analyses**

We considered correlated allele frequencies in the admixture model, using sampling locations as priors, and ran 10 iterations of 100,000 MCMC repetitions with 10,000 burn-in period (Gilbert et al. 2012; Janes et al. 2017). MCMC convergence, where α values reached equilibrium, was examined using the *Data plot* option in STRUCTURE (Porras-Hurtado et al. 2013). Variation of K values was then summarised and plotted in CLUMPAK (Kopelman et al. 2015).


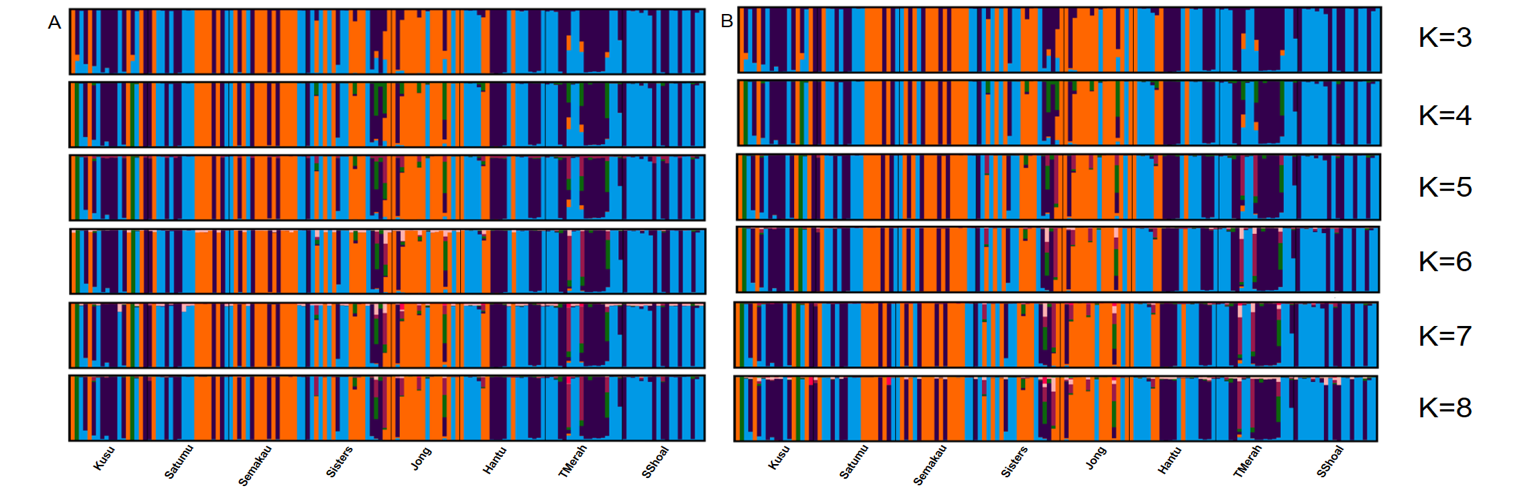


**Figure S6.** STRUCTURE plots K=3 to K=8 with (A) and without (B) putative loci under selection using 3649 SNPs and 3623 SNPs respectively, showing similar assignments of individuals to populations for *Porites* sp.


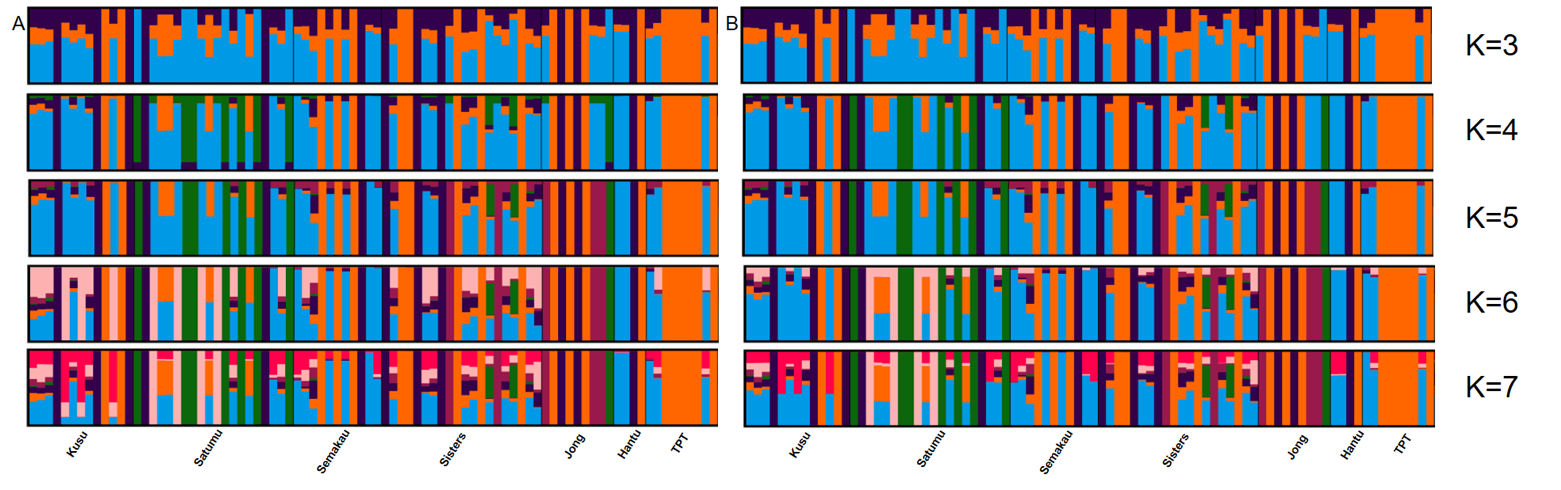


**Figure S7.** STRUCTURE plots of K=3 to K=7 with (A) and without (B) putative loci under selection using 5846 SNPs and 5813 SNPs respectively, showing similar assignments of individuals to populations for *Pocillopora acuta.*

**Evanno’s plots**

The optimal K was determined by examining the Ln Pr(X|K) and ΔK plots (Evanno et al., 2005; Janes et al., 2017)


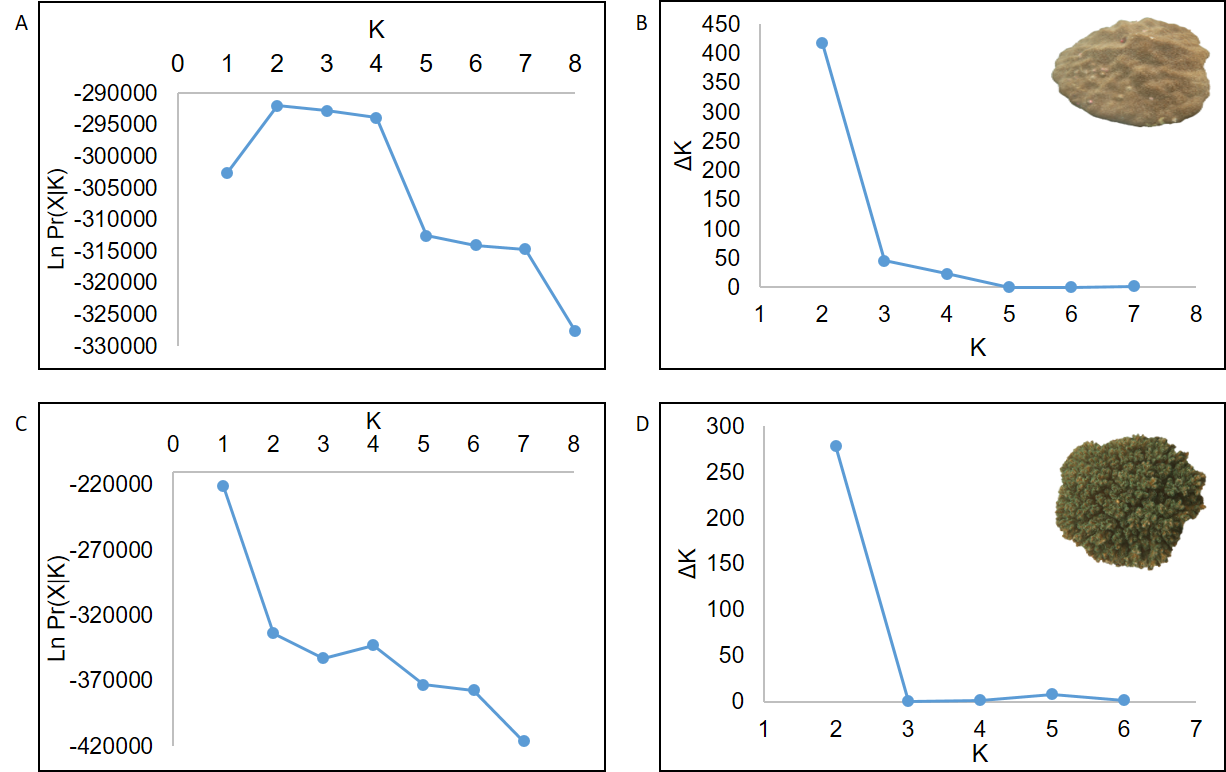


**Figure S8.** Evanno’s plots based on Ln Pr(X|K) and ΔK for *Porites* sp. (A, B) and *Pocillopora acuta* (C, D).


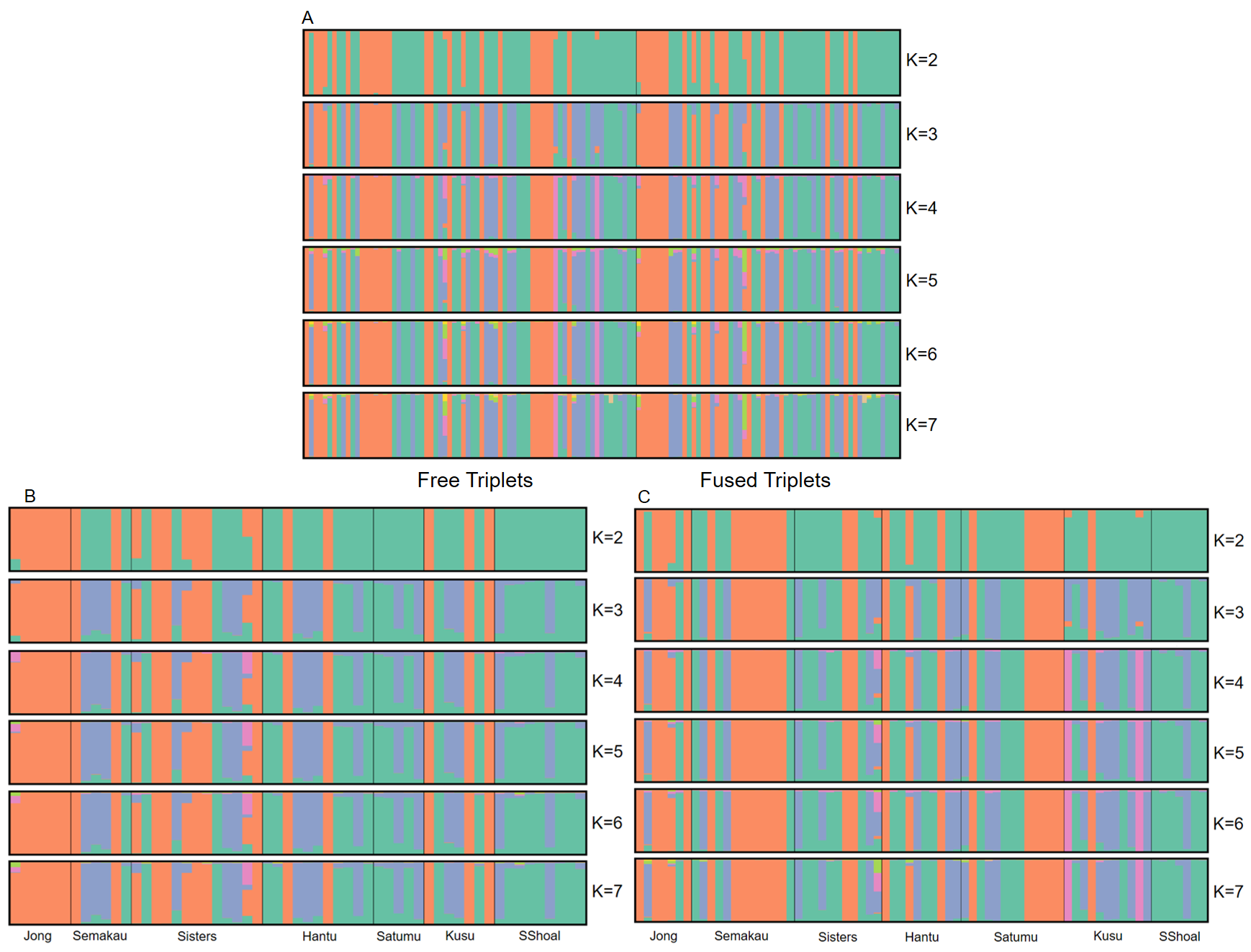


**Figure S9**: (A) STRUCTURE results of up to K=7 with individuals arranged according to ventral triplet formation of *Porites* sp. highlighting similar population genetic patterns between free and fused triplet colony forms. (B) & (C) show independent STRUCTURE analyses of free and fused triplet colony forms, respectively, also illustrating similar population genetic patterns and distribution of the three cryptic lineages.

**BayesAss migration rates**

We estimated contemporary gene flow in BayesAss v.3.0.4 (Wilson & Rannala, 2003) using 10,000,000 MCMC repetitions, a burn-in of 1,000,000 and a sampling interval of 1,000 iterations.

**
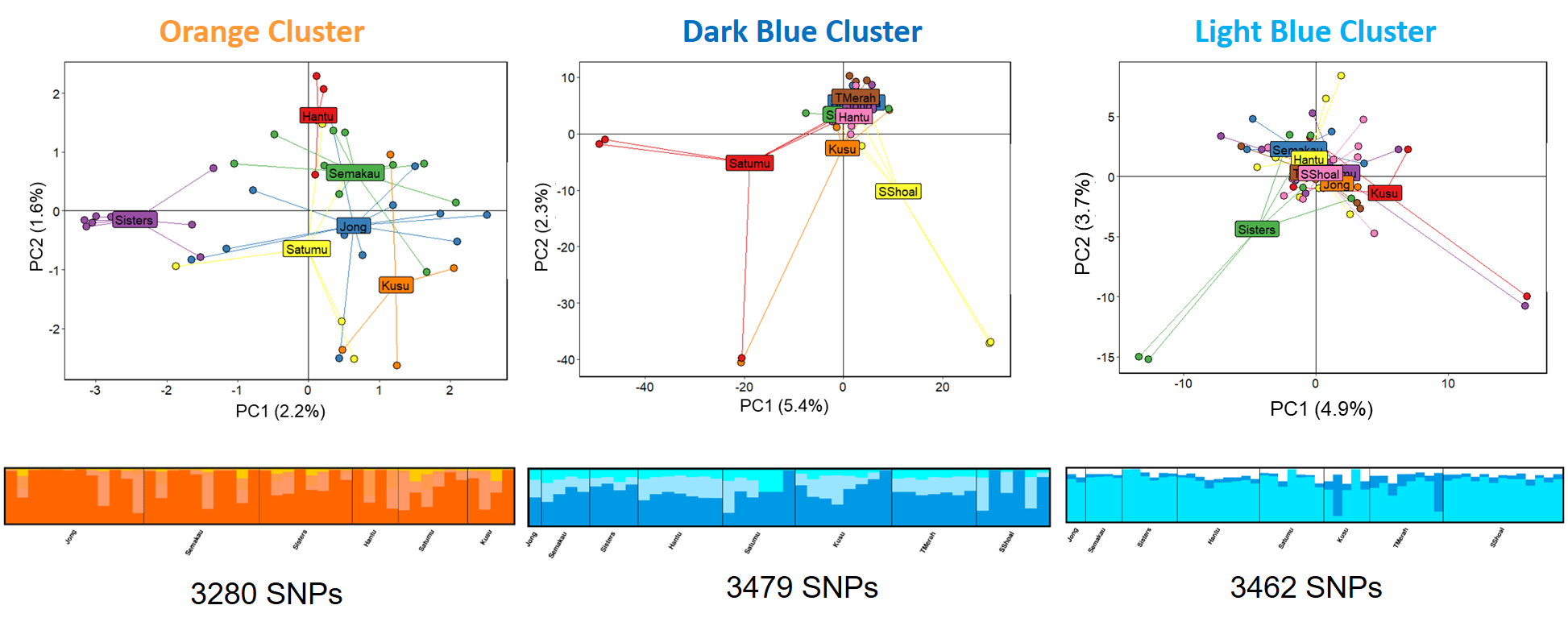
**

Figure S10: PCA and STRUCTURE (K=3) based on individual clusters of *Porites* sp indication panmixia within each cluster. Each of these clusters were independently used to estimate contemporary gene flow using BayesAss.

**Table S6.** Matrix of inferred (posterior mean) migration rates for *Porites* sp. taken over ten BayesAss runs with the standard deviation in parentheses. Proportion of migrants within sites (self-recruitment) are bolded. Other proportion of migrants that are illustrated in Figure 2C are underlined. Horizontal and vertical sites represent source and sink respectively. (A) Orange Cluster, (B) Dark Blue Cluster, (C) Light Blue Cluster.

(A)

| Sites | Kusu | Satumu | Semakau | Sisters | Jong | Hantu |
| --- | --- | --- | --- | --- | --- | --- |
| Kusu | **0.6825 (0.0132)** | 0.0176 (0.0165) | 0.0171 (0.0148) | 0.0158 (0.0150) | 0.0165 (0.0156) | 0.0157 (0.0151) |
| Satumu | 0.0198 (0.0091) | **0.6500 (0.0373)** | 0.0128 (0.0122) | 0.0186 (0.0120) | 0.0169 (0.0047) | 0.0057 (0.0021) |
| Semakau | 0.0168 (0.0110) | 0.0182 (0.0154) | **0.6125 (0.0142)** | 0.0159 (0.0151) | 0.0165 (0.0156) | 0.0159 (0.0152) |
| Sisters | 0.0920 (0.0103) | 0.0190 (0.0143) | 0.0467 (0.0132) | **0.5925 (0.0152)** | 0.0167 (0.0159) | 0.0161 (0.0153) |
| Jong | 0.0168 (0.0122) | 0.0677 (0.0169) | 0.0865 (0.0158) | 0.0759 (0.0153) | **0.8351 (0.0159)** | 0.0909 (0.0052) |
| Hantu | 0.0170 (0.0057) | 0.0169 (0.0152) | 0.0166 (0.0159) | 0.0157 (0.0150) | 0.0166 (0.0159) | **0.6226 (0.0152)** |

(B)

| Sites | Kusu | Satumu | Semakau | Sisters | Jong | Hantu | TM | SS |
| --- | --- | --- | --- | --- | --- | --- | --- | --- |
| Kusu | **0.6721 (0.0131)** | 0.0475 (0.0136) | 0.0166 (0.0139) | 0.0158 (0.0137) | 0.0065 (0.0035) | 0.0157 (0.0114) | 0.0392 (0.0117) | 0.0166 (0.0121) |
| Satumu | 0.0672 (0.0139) | **0.6431 (0.0220)** | 0.0136 (0.0109) | 0.0586 (0.0116) | 0.0069 (0.0024) | 0.0057 (0.0026) | 0.0158 (0.0021) | 0.0168 (0.0117) |
| Semakau | 0.0158 (0.0125) | 0.0175 (0.0075) | **0.6132 (0.0159)** | 0.0159 (0.0132) | 0.0265 (0.0121) | 0.0159 (0.0130) | 0.0159 (0.0128) | 0.0168 (0.0143) |
| Sisters | 0.0760 (0.0116) | 0.0175 (0.0086) | 0.0107 (0.0143) | **0.5925 (0.0124)** | 0.0067 (0.0063) | 0.0161 (0.0121) | 0.0159 (0.0106) | 0.0167 (0.0128) |
| Jong | 0.0159 (0.0137) | 0.0125 (0.0111) | 0.0565 (0.0128) | 0.0039 (0.0017) | **0.8351 (0.0127)** | 0.0131 (0.0156) | 0.0159 (0.0103) | 0.0165 (0.0104) |
| Hantu | 0.0159 (0.0119) | 0.0176 (0.0117) | 0.0166 (0.0117) | 0.0157 (0.0039) | 0.0166 (0.0045) | **0.7226 (0.0109)** | 0.0258 (0.0135) | 0.0379 (0.0185) |
| TM | 0.0843 (0.0218) | 0.0573 (0.0165) | 0.0098 (0.0075) | 0.0109 (0.0083) | 0.0031 (0.0036) | 0.0058 (0.0069) | **0.8136 (0.0308)** | 0.1367 (0.0213) |
| SS | 0.0800 (0.0195) | 0.0126 (0.0152) | 0.0170 (0.0123) | 0.0056 (0.0115) | 0.0039 (0.0025) | 0.0323 (0.0137) | 0.1313 (0.0157) | **0.8233 (0.0282)** |

(C)

| Sites | Kusu | Satumu | Semakau | Sisters | Jong | Hantu | TM | SS |
| --- | --- | --- | --- | --- | --- | --- | --- | --- |
| Kusu | **0.6525 (0.0142)** | 0.0426 (0.0149) | 0.0158 (0.0142) | 0.0162 (0.0142) | 0.0062 (0.0064) | 0.0162 (0.0101) | 0.0687 (0.0132) | 0.0067 (0.0136) |
| Satumu | 0.0651 (0.0127) | **0.5922 (0.0254)** | 0.0128 (0.0112) | 0.0593 (0.0120) | 0.0072 (0.0029) | 0.0059 (0.0021) | 0.0250 (0.0032) | 0.0172 (0.0123) |
| Semakau | 0.0169 (0.0142) | 0.0165 (0.0043) | **0.6239 (0.0140)** | 0.0164 (0.0129) | 0.0162 (0.0136) | 0.0163 (0.0122) | 0.0142 (0.0131) | 0.0175 (0.0154) |
| Sisters | 0.0730 (0.0149) | 0.0169 (0.0161) | 0.0141 (0.0151) | **0.5885 (0.0142)** | 0.0059 (0.0079) | 0.0157 (0.0119) | 0.0140 (0.0097) | 0.0171 (0.0146) |
| Jong | 0.0163 (0.0147) | 0.0114 (0.0127) | 0.0557 (0.0132) | 0.0043 (0.0127) | **0.8220 (0.0168)** | 0.0136 (0.0162) | 0.0139 (0.0142) | 0.0173 (0.0138) |
| Hantu | 0.0167 (0.0101) | 0.0161 (0.0092) | 0.0159 (0.0159) | 0.0161 (0.0147) | 0.0066 (0.0039) | **0.7230 (0.0136)** | 0.0264 (0.0148) | 0.0565 (0.0206) |
| TM | 0.0796 (0.0230) | 0.0542 (0.0170) | 0.0091 (0.0074) | 0.0790 (0.0109) | 0.0027 (0.0121) | 0.0064 (0.0112) | **0.8021 (0.0293)** | 0.1402 (0.0213) |
| SS | 0.0621 (0.0195) | 0.0419 (0.0161) | 0.0220 (0.0137) | 0.0048 (0.0118) | 0.0043 (0.0048) | 0.0623 (0.0148) | 0.1480 (0.0175) | **0.8129 (0.0301)** |

**Table S7.** Matrix of inferred (posterior mean) migration rates for *Pocillopora acuta* taken over ten BayesAss runs with the standard deviation in parentheses. Proportion of migrants within sites (self-recruitment) are bolded. Horizontal and vertical sites represent source and sink respectively.

| Sites | Kusu | Semakau | Sisters | Jong | Hantu | TPT |
| --- | --- | --- | --- | --- | --- | --- |
| Kusu | **0.7329 (0.0221)** | 0.0228 (0.0216) | 0.0575 (0.0286) | 0.0154 (0.0307) | 0.0226 (0.0325) | 0.0187 (0.0179) |
| Semakau | 0.0188 (0.0181) | **0.7476 (0.0267)** | 0.0115 (0.0107) | 0.0193 (0.0183) | 0.0221 (0.0232) | 0.0100 (0.0200) |
| Sisters | 0.1267 (0.0229) | 0.0427 (0.0209) | **0.6792 (0.0229)** | 0.0182 (0.0179) | 0.0162 (0.0204) | 0.0100 (0.0233) |
| Jong | 0.0167 (0.0137) | 0.0169 (0.0165) | 0.0484 (0.0186) | **0.7012 (0.0302)** | 0.0255 (0.0236) | 0.0180 (0.0181) |
| Hantu | 0.0150 (0.0142) | 0.0347 (0.0219) | 0.0124 (0.0106) | 0.0190 (0.0181) | **0.6421 (0.0352)** | 0.0580 (0.0169) |
| TPT | 0.0525 (0.0217) | 0.0657 (0.0289) | 0.0170 (0.0236) | 0.0682 (0.0290) | 0.0526 (0.0309) | **0.6360 (0.0320)** |

**References**

Brakel, W. H. (1977). Corallite variation in Porites and the species problem in corals. In *Proceedings of the Third International Coral Reef Symposium* (Vol. 1, pp. 457-462).

Evanno G., Regnaut S., Goudet J. (2005) Detecting the number of clusters of individuals using the software STRUCTURE: a simulation study. *Molecular Ecology* 14:2611–20

Excoffier L., Lischer H. E. L. (2010) Arlequin suite ver 3.5: A new series of programs to perform population genetics analyses under Linux and Windows. *Molecular Ecology* *Resources* 10:564–567

Foll M., Gaggiotti O. (2008) A genome-scan method to identify selected loci appropriate for both dominant and codominant markers: A Bayesian perspective. *Genetics* 180:977–993

Forsman, Z., Wellington, G. M., Fox, G. E., & Toonen, R. J. (2015). Clues to unraveling the coral species problem: distinguishing species from geographic variation in Porites across the Pacific with molecular markers and microskeletal traits. *PeerJ*, *3*, e751.

Gilbert K. J., Andrew R. L., Bock D. G., Franklin M. T., Kane N. C., Moore J. S., Moyers B. T., Renaut S., Rennison D. J., Veen T., Vines T. H. (2012) Recommendations for utilizing and reporting population genetic analyses: The reproducibility of genetic clustering using the program structure. *Molecular Ecology* 21:4925–4930

Huang, D., Tun, K. P. P., Chou, L. M., & Todd, P. A. (2009). An inventory of zooxanthellate scleractinian corals in Singapore , including 33 new records. *The Raffles Bulletin of Zoology*, (22), 69–80.

Jameson, S. C., & Cairns, S. D. (2012). Neotypes for Porites porites (Pallas, 1766) and Porites divaricata Le Sueur, 1820 and remarks on other western Atlantic species of Porites (Anthozoa: Scleractinia). *Proceedings of the Biological Society of Washington*, *125*(2), 189-207

Janes J. K., Miller J. M. , Dupuis J. R. , Malenfant R. M., Gorrell J. C., Cullingham C. I., Andrew R. L. (2017) The K = 2 conundrum. *Molecular Ecology* 3594–3602

Knowlton, N., Mate, J. L., Guzman, H. M., Rowan, R., & Jara, J. (1997). Direct evidence for reproductive isolation among the three species of the Montastraea annularis complex in Central America (Panama and Honduras). *Marine Biology*, *127*(4), 705-711.

Kopelman N. M., Mayzel J., Jakobsson M., Rosenberg N. A., Mayrose I. (2015) CLUMPAK: A program for identifying clustering modes and packaging population structure inferences across K. *Molecular Ecology* *Resources* 15:1179–1191

López-Pérez, R. A. (2013). Species composition and morphologic variation of Porites in the Gulf of California. *Coral reefs*, *32*(3), 867-878.

Poquita-Du, R. C., Quek, Z. B. R., Jain, S. S., Schmidt-Roach, S., Tun, K., Heery, E. C., … Huang, D. (2019). Last species standing: loss of Pocilloporidae corals associated with coastal urbanization in a tropical city state. *Marine Biodiversity*. <https://doi.org/10.1007/s12526-019-00939-x>

Poquita-Du, R., Ng, C. S. L., Loo, J. Bin, Afiq-Rosli, L., Tay, Y. C., Todd, P., … Huang, D. (2017). New evidence shows that Pocillopora ‘damicornis-like’ corals in Singapore are actually Pocillopora acuta (Scleractinia: Pocilloporidae). *Biodiversity Data Journal*, *5*, e11407. <https://doi.org/10.3897/BDJ.5.e11407>

Schneider C.A., Rasband W.S., Eliceiri K.W. (2012) NIH Image to ImageJ: 25 years of image analysis. Nature Methods, 9, 671–675.

Stefani, F., Benzoni, F., Pichon, M., Mitta, G., & Galli, P. (2008). Genetic and morphometric evidence for unresolved species boundaries in the coral genus Psammocora (Cnidaria; Scleractinia). *Hydrobiologia*, *596*(1), 153-172.

Tisthammer, K. H., & Richmond, R. H. (2018). Corallite skeletal morphological variation in Hawaiian Porites lobata. *Coral Reefs*, 1-12.

Todd, P. A., Sidle, R. C., & Lewin-Koh, N. J. I. (2004). An aquarium experiment for identifying the physical factors inducing morphological change in two massive scleractinian corals. *Journal of Experimental Marine Biology and Ecology*, *299*(1), 97-113.

Veron, J. E. N. (1982). Scleractinia of eastern Australia, Part IV, Family Poritidae. *Australian Institute Marine Science, Monograph Series* *5*, 159p.

Veron, J. E. N. (2000). Corals of the World, vol. 3. *Australian Institute of Marine Science, Townsville*, *295*.

Weil, E. (1992, June). Genetic and morphological variation in Caribbean and eastern Pacific Porites (Anthozoa, Scleractinia). Preliminary results. In *Proc 7th int coral Reef Symp* (Vol. 2, pp. 643-656).

Wilson G. A., Rannala B. (2003) Bayesian inference of recent migration rates using multilocus genotypes. *Genetics* 163:1177–1191

Wong, J. S. Y., Chan, Y. K. S., Ng, C. S. L., Tun, K. P. P., Darling, E. S., & Huang, D. (2018). Comparing patterns of taxonomic, functional and phylogenetic diversity in reef coral communities. *Coral Reefs*, *37*(3), 737–750. https://doi.org/10.1007/s00338-018-1698-6
